# The benefits of mind wandering on a naturalistic prospective memory task

**DOI:** 10.1038/s41598-023-37996-z

**Published:** 2023-07-15

**Authors:** J. C. Girardeau, R. Ledru, A. Gaston-Bellegarde, P. Blondé, M. Sperduti, P. Piolino

**Affiliations:** 1 Laboratoire Mémoire, Cerveau & Cognition (LMC2 UR 7536), Institut de Psychologie, Université Paris Cité, 71 Ave Édouard Vaillant, 92100 Boulogne-Billancourt, France; 2 Icelandic Vision Lab, University of Iceland, Reykjavik, Iceland

**Keywords:** Psychology, Human behaviour

## Abstract

Mind wandering (MW) occurs when our attention spontaneously shifts from the task at hand to inner thoughts. MW is often future-oriented and may help people remember to carry out their planned actions (Prospective Memory, PM). Past-oriented MW might also play a critical role in boosting PM performance. Sixty participants learned 24 PM items and recalled them during an immersive virtual walk in a town. The items were divided into event-based—EB and time-based—TB. During the PM retention phase, participants were randomly assigned to a high or a low cognitive load condition, in order to manipulate MW frequency. Some PM items were encoded before this MW manipulation (pre-PM) and some during the virtual walk (post-PM). A high MW frequency was linked with better global PM performances. Spontaneous past-oriented MW predicted better pre-EB retrospective PM retrieval, while spontaneous future-oriented MW predicted better Pre-EB prospective PM retrieval. Voluntary future-oriented MW predicted better post-EB retrospective retrieval. We highlighted, for the first time, a differential impact of spontaneous MW content depending on the PM component (retrospective or prospective). Past‐oriented MW is crucial for (re)consolidating PM intentions, and episodic future thinking MW for the execution of PM intentions. We discuss the twofold functional role of MW, namely, to consolidate an already programmed intention and to plan future actions.

## Introduction

Have you ever been in the situation where, during a conference talk, you realize that you cannot remember a word the keynote speaker said because you were thinking about your personal issues? This fundamentally human mental state that arises from our inner stream of consciousness is called mind wandering (MW) and occurs when your mind stops being in the present moment. Generally, MW reflects the disengagement of attentional focus from the environment toward self-generated thoughts unrelated to the activity at hand^1–3^. This cognitive state has received, in the last decade, great scientific interest as MW is a prominent phenomenon, occupying between 30 and 50% of our waking mental activity^2–7^.

Contrary to what this description of MW suggests, its operationalization and theoretical definition are the subject of lively debate^4–6^. Some authors argue that MW is above all characterized by its dynamical and unconstrained evolution^7^, while others consider that its definition rests mainly on its content, which can be: task-unrelated^8^ or stimulus-independent thoughts^9^. Some authors even propose a distinction between spontaneous and voluntary MW^3^, the former corresponding to an involuntary switch from a task-related focus to a task-unrelated one, while the latter is a conscious choice to stop paying attention to the task at hand. Given that there is no consensus on a single operational definition to date, Seli et al. (2018) proposed to consider this concept from the perspective of a “Family Resemblance View”, in which the different existing definitions of MW are seen as different complementary aspects of the phenomenon^9,10^. Its attributes may be shared by different definitions, or be specific to some, with the most widely used attributes characterizing the most prototypical definition of MW.

So far, MW has been mainly associated with major cognitive disagreements^3,10–13^. The overall consensus is that it has a broadly negative impact, hindering focused attention^14,15^, working memory capacity^16,17^ and episodic memory encoding^4^ to name a few effects. However, it would be surprising if such a prevalent phenomenon served no purpose. A series of studies has opened a new avenue of interest regarding its functional role^13^. As previously mentioned, the initiation of an MW episode can be triggered in two different ways: spontaneously or voluntarily. Spontaneous MW is associated with certain measures of mindfulness such as a greater tendency to perform actions without being aware of them^18^. In contrast to involuntary MW, intentional episodes of MW are strongly associated with planning and awareness of the initiation of the episode of MW, resulting in a meta-cognitive awareness of its occurrence^15^. The voluntary shift in attentional focus and redirection of these resources to internal thought processing that deliberate MW is believed to induce^9,19,20^ could therefore be likely to strengthen the processes of anticipating, developing and planning goals associated with individuals’ current preoccupations^21–23^. The internal stimuli on which attention is focused during an MW instance are not necessarily decontextualized thoughts (general reflections, internal questioning, etc.). On the contrary, they can be temporal, whether they are about the past, the present or the future. In other words, mind wandering can frequently coincide with instances of mental time travel. A general finding from a growing number of studies has acknowledged the existence of a “prospective bias” in MW content^7,20,24^. That is, off-task thoughts are more frequently categorized as being about the near future (today—tomorrow) and goal-directed. These data suggest that when people think about the future in their daily lives, they frequently think about their upcoming planned actions^25,26^. The idea that mind wandering may serve the pursuit of our prospective goals has been discussed for a long time^19,27,28^. Stawarczyk et al.^29^ already pointed to the role of inner speech in the mental representation of prospective intentions in order to better plan their formulation^19,20,30^, particularly through the internal enunciation of the future action. A recent review confirmed that future-oriented thoughts are mainly spontaneous, focused on the near future and concerned with concrete goals (i.e. planned intentions) rather than hypothetical projections (i.e. episodic future thinking)^31^. Nevertheless, even if the temporal orientation of wandering thoughts has been repeatedly shown to be primarily focused on the future, either in the context of planning actions to be performed or projecting into hypothetical situations^32–35^, some observations do not highlight such a bias and emphasize that a large part of mind wandering is dedicated to the recall of past events^36^. Although the literature on the impact of mind wandering on the consolidation of new information is still underdeveloped, several studies have already investigated how past-oriented MW content is associated with the (re)consolidation of older memories, mainly via mental imagery that allows the refreshing of previous experiences^36–38^. Thus, mental imagery may contribute to binding all the context surrounding the realization, at the appropriate moment, of the prospective action, via the mental projection of the realization of the intention and the visual retrieval of the encoding source. MW could therefore promote consolidation in episodic memory via the implementation of re-encoding and be, in the long term, beneficial for information already present in episodic memory. Since the representations of unfulfilled intentions are more accessible to memory retrieval^24,27,28,39,40^, the number of MW episodes associated with the retrospective content of a upcoming task may increase during the delay interval or the retention phase of a prospective memory task^30,41–43^. Thus, the reactivation of intention throughout an MW episode could allow its re-encoding, which would strengthen the intention’s representation, leading to a better intention retrieval. MW could thus have the potential role of disengaging cognitive resources from the here-and-now in order to plan or execute previously planned actions, a milestone of what is known as prospective memory. This question remains open and, while a period of rest has been repeatedly shown to consolidate information encoding^44^, whether this effect can be linked to MW has been little studied. More broadly, and with respect to our study, there are even fewer studies that have directly tested the relationship between the propensity to MW and PM.

Prospective memory (PM) refers to the ability to remember to execute previously formulated intentions, after a variable interval occupied by an ongoing activity^31,45,46^. Unlike retrospective memory, on which PM in part relies, PM does not simply store memories or knowledge per se, but rather the intention to perform an action at the appropriate time (*time-based*) or in the appropriate context (*event-based*). This means that the information stored in PM is twofold, including the intention (e.g., I have to buy bread) and the moment when it must be executed (e.g., when coming home from work). Thus, PM comprises two components: the prospective component corresponds to the planning of the intention (e.g., the time and the context related to the recall of the action, the WHEN and WHERE); the retrospective component refers to the content of the intention, to the precise action that has to be carried out (the WHAT). Once encoded, according to Ellis and Kvavilashvili^47^, the PM intention is then stored in retrospective memory and is no longer activated in working memory^32,33^. To sum up, from planning to the execution of an intention, the whole PM process is underpinned by various cognitive functions and requires attentional and executive resources^33–35,48–51^. Beyond the essential aspect of a good functioning of executive and attentional functions for an efficient PM, what happens during the retention delay remains rather understudied. Between the establishment of an intention (e.g., I have to buy bread) and the moment at which it must be executed (e.g., when coming home from work), the intention is backgrounded for a variable length of time. During this period, ongoing activities may capture the person’s attention and interfere with the detection of the PM cues^31,33,52^. On the contrary, some activities intervening during this period could potentially favour both the retrospective and prospective components of PM. Thus, when the mind momentarily wanders, the train of thought could offer a unique opportunity for a latent PM goal, according to attentional demand, to become active in the mind and to be consolidated or accomplished^53^.

To our knowledge, few studies have directly investigated the links between MW and PM^26,54,55^. Using voluntary MW as a control condition compared to a mindfulness meditation exercise, one of our previous studies^51^ reported no difference between these two supposedly opposed cognitive states (i.e., mindfulness and mind wandering) on PM, the former being assumed to have a positive impact on PM performance. However, results showed that the two inductions subjectively had a differential effect on the participants’ cognitive state. It was speculated that MW could have a similar beneficial effect on PM performance via a transient increase in PM monitoring. Another possible explanation is that the prospective bias of MW could have boosted PM performances. Nevertheless, and contrary to this theoretical proposal, we reported in an experience sampling study^26^ did not report an association between the propensity to future-oriented MW and PM performance^7^. Surprisingly, a link was observed instead between past‐oriented thoughts and the execution of a prospective intention. These results seem to be consistent with a functional role of past-oriented MW, as it may serve to consolidate an already programmed intention. This might make sense if one considers that if the MW occurs during the PM retention period, this would be a potential opportunity to consolidate a prospective intention. However, due to the methodology employed in this naturalistic study, in which MW and PM were assessed separately, we were unable to test this hypothesis directly. This interpretation therefore remains largely speculative. The aim of the present study was to test this hypothesis in a more controlled laboratory-based paradigm, specifically manipulating MW during the PM retention phase. From a theoretical point of view, it has been proposed that MW engages executive resources, and thus that manipulating on-going task-demand should affect the rate of MW (e.g., the higher the task-demand, the lower the resources available for MW)^12,56^. This prediction has been confirmed by numerous studies showing that more difficult tasks, irrespective of their nature (e.g., visual processing, reading, driving^44,57–60^), were accompanied by lower rates of MW, compared to easier ones. We capitalized on these findings to manipulate the MW rate by varying the difficulty of a working memory task during the retention interval.

The objective of this study was to investigate the causal link between MW and PM. More specifically, we tested the differentiated impact of MW temporal orientation (past or future) during the retention interval on PM performances, in an ecological setting. We wanted to replicate our previous findings concerning the predictive role of past-oriented thoughts on PM performances^26^, by increasing experimental control of the MW frequency during the PM retention phase^61^. If this effect is indeed based on the consolidation process, this should be true only for those PM intentions encoded before MW induction, but not for those newly encoded during the PM task. Therefore, in this study, some prospective intentions were encoded before the encoding phase while others were proposed “on-line”, during the PM task.

We chose to use virtual reality (VR) as on optimal compromise between traditional laboratory PM tasks and real life-based approaches, which have given rise to heterogeneous results. The former lack ecological validity, while the latter are particularly far from the situations encountered in daily life^62^. Faced with these issues, VR has emerged as a promising solution for evaluating PM^63–65^. However, there are different levels of immersion and realism of interaction with the environment in VR tasks. While immersion and sense of presence are considered along a spectrum, VR systems are frequently divided into non-immersive and immersive systems. Non-immersive VR refers to virtual environments projected onto two-dimensional displays (i.e., hardware) such as computer screens, laptops, tablets, and mobile devices^66–68^, whereas immersive VR refers to virtual environments projected onto devices such as head-mounted displays (HMDs) and CAVE systems^66–69^. Non-immersive VR provides a less ecologically valid testing environment than immersive VR tests^70–73^. An immersive VR research paradigm provides the most efficient approach to assess PM in naturalistic and interactive conditions promoting a sense of presence^74,75^. By placing subjects in a multitude of daily life situations for which they can recall a wide variety of intentions, it simulates the complexity of the activities of daily life while maintaining experimental rigor, to obtain a measurement that is both sensitive, complete and specific to the functioning of the PM and its various components.

## Materials and methods

### Participants

This study was conducted on 60 participants (mean age: 22.08 ± 4.76 years). Participants were recruited either through Université Paris Cité intranet or through RISC (Relay of information on the sciences of cognition). For Université Paris Cité students, participants received credits to validate a course on research methodology. On the RISC platform, voluntary participation in the experiment was compensated by a 10-euro voucher. To be eligible for the study, subjects should: (1) be between 18 and 35 years old, (2) be a French speaker, (3) have normal or corrected vision, (4) not have sensorimotor deficits incompatible with virtual reality navigation, (5) not have a history of neurological or psychiatric disorders, (6) not be taking medication that could affect cognitive functioning, (7) not be easily prone to motion sickness, (8) not be at risk of developing a severe form of Covid-19 disease, (9) not be a regular/expert meditation practitioner. In virtual reality (VR), participants may be subject to nausea related to navigation in a VR environment. This phenomenon, called cybersickness, is more pronounced in individuals prone to motion sickness. This exclusion criterion therefore allowed us to exclude participants who could suffer from this illness during the experiment and thus to reduce the dropout rate of the subjects in the study. The last exclusion criterion concerning the practice of meditation is also important as people who are qualified as “experts” in meditation (i.e. several hours per week for several years), will tend to limit their flow of wandering thoughts more, which could constitute a bias in our study^76,77^. Informed consent was obtained from all participants, and the ethical committee from University Paris City approved the study which has been performed in accordance with the Declaration of Helsinki.

Each participant was randomly assigned to one of the following two groups: Low Cognitive Load (N = 30, 24 females and 6 males, mean age = 20.96 ± 3.5 years) and High Cognitive Load (N = 30, 27 females and 3 males, mean age = 22.76 ± 5.23) according to a between-subject design. The cognitive load was manipulated in order to induce more (Low Cognitive Load) or less (High Cognitive Load) MW frequency^71^. The “low cognitive load” group will be referred to as the EASY group for the rest of the study, while the “high cognitive load” group will be referred to as the HARD group.

Subjects were matched on demographic (age, t(58) = − 1.57, *p* = 0.12; academic level, t(58) = − 1.57, *p* = 0.12), anxiety and depression (PHQ-4^78^, t(58) = 0.316, *p* = 0.75; anxiety, t(58) = 1.81, *p* = 0.75 and depression; t(58) = 0.32, *p* = 0.75), executive function abilities such as inhibition (Stroop–Stroop, 1935; t(58) = 1.7, *p* = 0.09), flexibility (Switch^79^; t(58) = 1.51, *p* = 0.136), updating in working memory (SimAct^79^; t(58) = − 0.127, *p* = 0.9) and metamemory aptitude (MPMI-s^80^; t(58) = − 0.445, *p* = 0.658). They were also matched on mind wandering trait (MWQ^26^; t(58) = 1.73, *p* = 0.09). Descriptive statistics are reported in Supplementary Materials (Table 2).

### Materials

The computer equipment for the tests and questionnaires, as well as for the MW task, consisted of a Dell monitor with a resolution of 1920 × 1200 pixels, a keyboard and a mouse. The set of cognitive questionnaires and tests, as well as the MW task was programmed in Python 3.6, using the open-source module Neuropsydia 1.0.5^81^.

The VR equipment used for immersion and interaction in the virtual environment was the HTC Vive PRO set. It was composed of a VR headset with integrated headphones, two controllers, present on both sides of the headset on the image, as well as two spatial sensors in the background. Placed in two opposite corners in the VR room, the sensors make it possible to locate at any time the helmet and the controllers in space to allow navigation. The STEAMVR™ software can play any VR game especially with the HTV Vive Pro headset used here. The VR environment used for the experiment was entirely designed using UNITY v2021.1.20 software.

**Figure 1:**
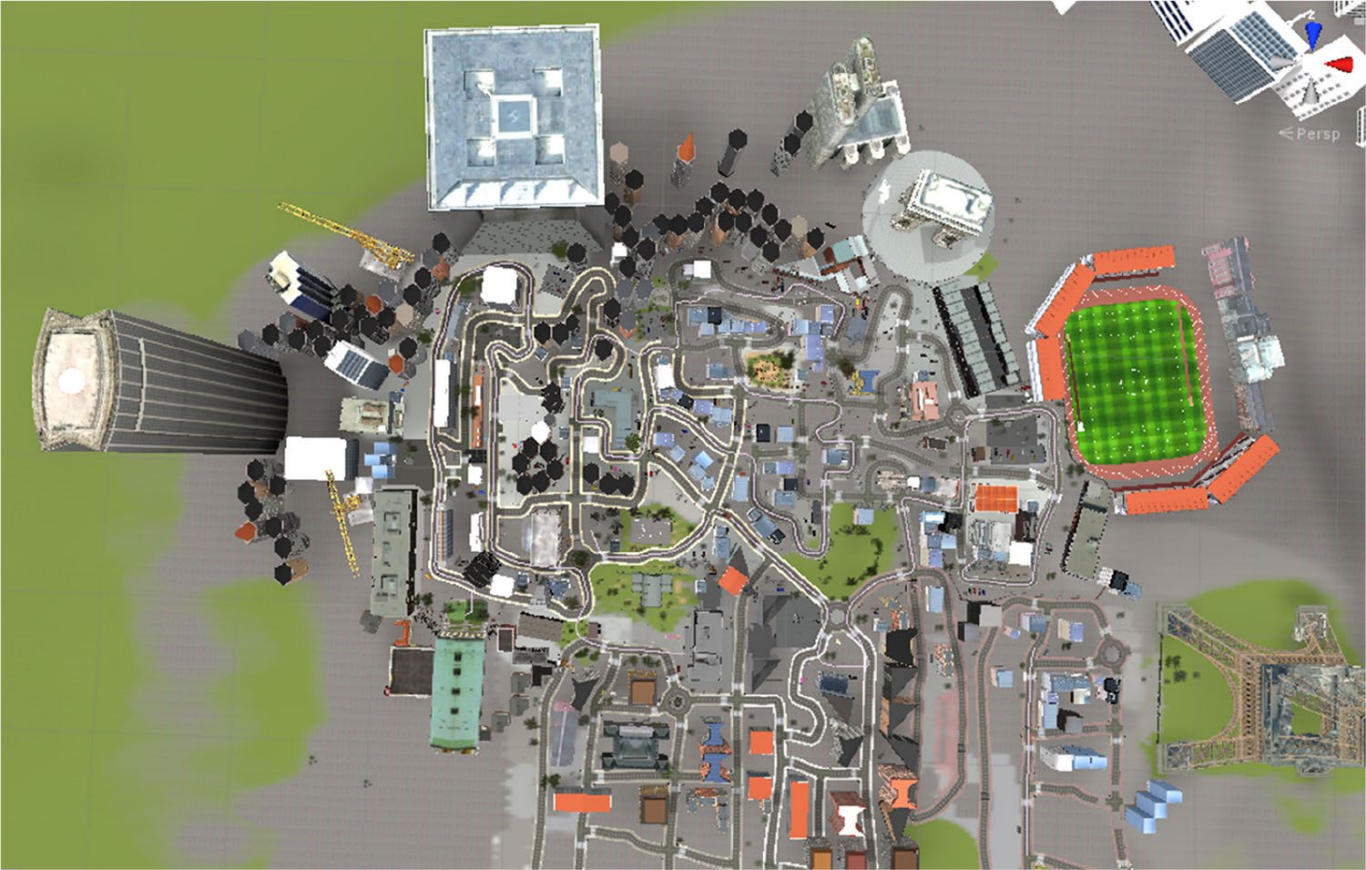
Aerial view of the virtual city designed in the MC^2^ Lab using UNITY v2021.1.20 software.

The virtual environment (VE), developed at the MC^2^ Lab (e.g., https://osf.io/x3m5z), consisted of a large city resembling Paris, with roads, cars, sidewalks with passers-by and animated scenes with avatars as well as buildings, stores, gas stations, parks, and famous places such as the Eiffel Tower and the arch of La Défense (Fig. 1). A soundtrack reproduces ambient city noise (horns, conversations of passers-by, etc.) and the interaction of the subject in VR is also represented in the VE in real time (appearance of verbal instructions, audio tapes, …). An environment specific to the training phase (presented next) was also created. It was similar to the city of the main task, but differed slightly in terms of the buildings encountered, the shops, monuments, etc.

### Mind wandering manipulation and assessment

**Figure 2:**
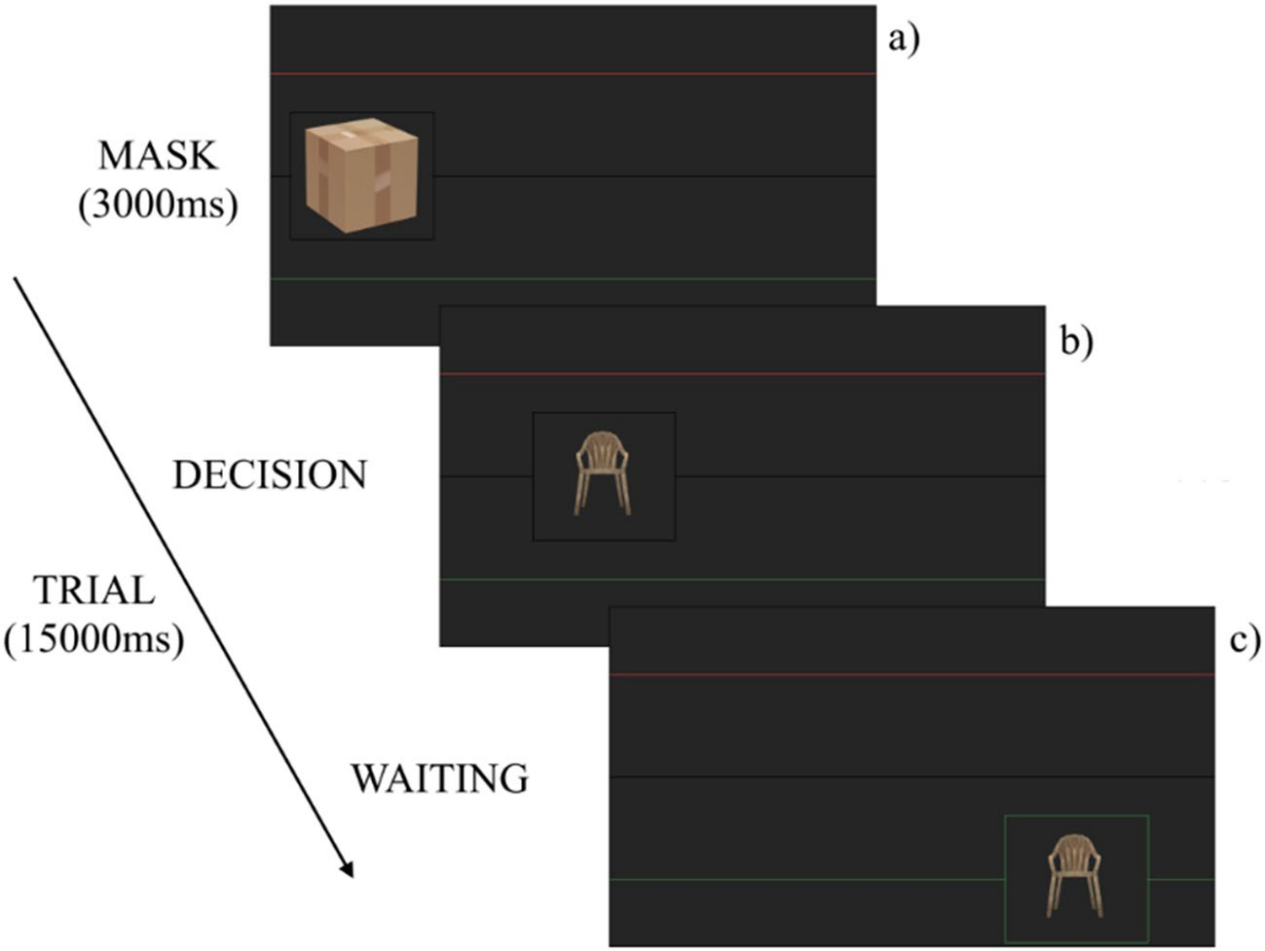
Schematic representation of a trial. The item drifted slowly from left to right along the black line, first masked as a cardboard box for the first 3 s of the trial (**a**). Then, when it appeared, the participant had to redirect it to one of the colored lines (**b**). Afterward, the item could not be moved until the end of the trial, for a total duration of 15 s (**c**).

The MW manipulation was the same as that used in our previous study^71^. The task included two blocks (Easy & Hard), each participant being randomly assigned to one of them. Each block was composed of 48 trials presented in a random order. In each block, one item (the image of a common object such as a tool, a piece of furniture, or food) was presented on-screen. Of these 48 trials, 42 presented different items (lures), while the remaining six presented six previously seen items (target) which were randomly selected for each participant. The items moved slowly (at a speed of about 3.5 cm/s) from left to right along a black line in the centre of the screen, as if they were moving along a factory line (Fig. 2). To reinforce this impression—and to serve as a mask—the image was not visible at first, hidden by the image of a cardboard box, until it reached the first tier of the line (after about 3 s), at which point the box disappeared and revealed the item. This was done to avoid the participant processing the item before it was fully visible on the screen. Two parallel lines were also present on the screen: a red and a green one, respectively above and below the central line. Both were equidistant from the black line (7.5° of visual angle, at a distance of 60 cm). Participants were informed that they had to perform a classification task on the objects and were told that the task was like “being a factory worker processing items on a line”. More precisely, they had to perform an adaptation of the n-back task, where they had to compare the image presented on the screen to the previous image (1-back, low working memory load condition, EASY) or the image presented three trials before (3-back, high working memory load condition, HARD). If the two images matched, participants had to move the item, as quickly as possible, up to the red line, with the up key. If not, the image had to be moved down to the green line, with the down key. After the participant’s response, the images stayed on-screen until the end of the trial (for a total of about 15 s). Participants were also informed that, from time to time, a thought probe would appear on the screen and temporarily stop the task.

In each condition, twelve probes were randomly presented. Each probe was made up of a sequence of several questions. The questions asked were progressive and conditional to the answers to the previous questions (Table 1). In the first question, participants had to indicate the object of their attention: “I’m totally focused on the task” (***Focus***), “I’m totally focused on my thoughts” (***Mind wandering***) or “I was distracted by an external event” (***External***). Questions related to the MW response assessed both the type of MW (*Episodic Past Thinking (EPT), Episodic Future Thinking (EFT), Planning, Imaginary or Other*) and the source of MW (*Spontaneous* or *Voluntary*). Probes were dispatched pseudo randomly, every two to four trials (30–60 s) and stayed on-screen until the participant’s response. Both conditions lasted for about 15 m.

**Table 1:** Mind wandering probes: Questions and their responses.

*Q1—Indicate the object of your attention*	*I am totally focused on the task at hand—****Focus*** *I am totally focused on my thoughts—****Mind wandering (MW)*** *I was distracted by an external event (noise, visual, sensation, *etc.*…)—****External***
***Focus questions***	
*Q2—Did this require an effort? ****conditioned on the answer “Focus” to Q1***	*Yes, No*
*Q3—Do you think you can maintain your focus over time?**** conditioned on the answer “Yes” to Q2***	*Yes, No*
***MW questions***	
*Q4—Was it voluntary?**** conditioned on the answer “MW” to Q1***	*Yes, No*
*Q5—What exactly were you thinking about?**** conditioned on the answer “Yes” to Q4***	*I was thinking of a past event (last holiday, evening with friends, exam or meeting, *etc*.)–****Episodic Past Thinking—EPT*** *I was thinking about the future (next holiday, life goals, *etc*.)—****Episodic Future Thinking—EFT*** *I was thinking about something specific I need to remember to do in the future (appointment, mail, shopping, *etc*.)—***Planning** *I was thinking of an imaginary situation (fictive, unreal)—****Imagination*** *I wasn't thinking about anything in particular, nor was I focused on the current task—****Other***
***External questions***	
*Q6—Was it voluntary?**** conditioned on the answer “External” to Q1***	*Yes, No*
*Q4—Was your attention focused on this object?**** conditioned on the answer “Yes” to Q6***	*Yes, No*

### Prospective memory assessment

#### Encoding

Using a slide show broadcast on a computer screen, participants were invited to memorize a list of 15 items, consisting of associations between a cue (a picture) and an action (a sentence), presented in a different order from that in which they will be recalled. The items were divided into 3 categories (5 items per category): *event-based* semantically linked—EBL (e.g., “At the florist’s, buy a plant”); *event-based* semantically non-linked—EBN (e.g., “At the gas station, buy ice-cubes”) and *time-based—*TB (e.g., “At 5 min, drink some water”). Participants had to read aloud the content of the slide presented to them, i.e., a sentence linking the cue to the action (example: “At the market, buy vegetables”). Participants had as much time as they wanted to memorize it. Once they had moved on to the next slide, they could not go back. At the end of the 15 items of the list, a cued recall was carried out. For each of the 15 items, participants were shown the spatio-temporal cue, and they had to recall the associated action. If they failed to recall the entire list, they could repeat learning for up to three tries. Each error was reported on a rating sheet.

Nine extra PM items (3 EBL, 3 EBN and 3TB) were also presented during the virtual city navigation, through interactions with other avatars or reception of virtual SMS.

#### Virtual environment training

**Figure 3:**
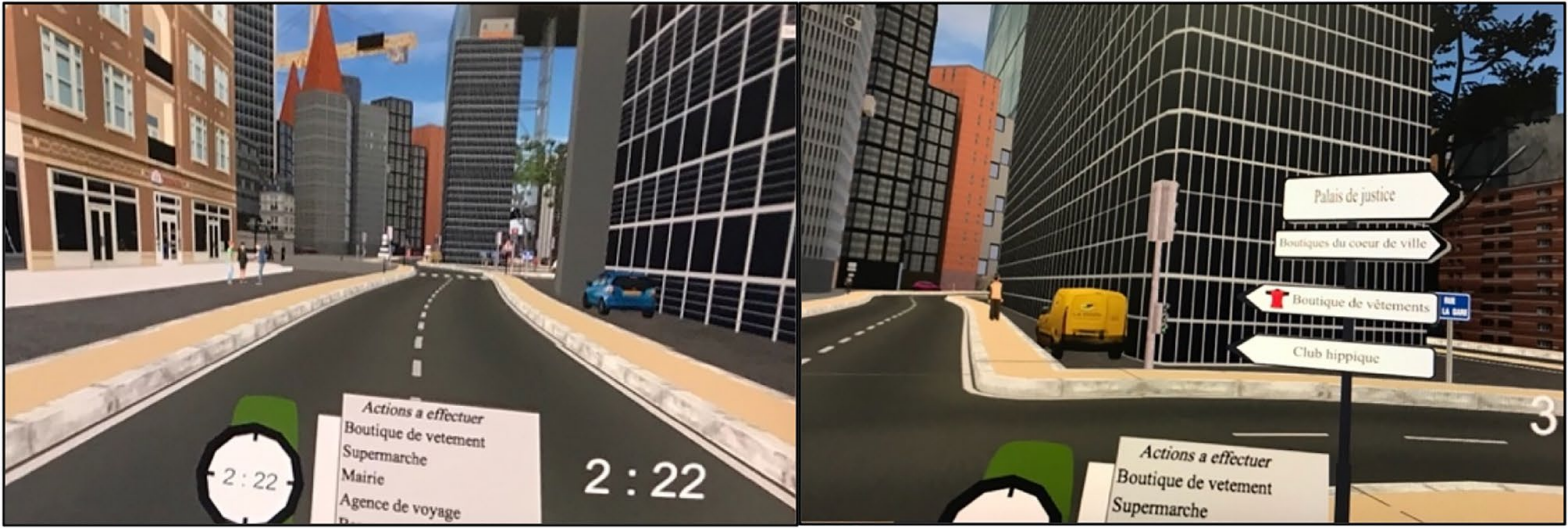
Virtual watch with the list of items to reach in the city and an example of an orientation sign.

The workout was progressive and divided into five sequences. The objective was to teach the subject, in a sequential way, to carry out all four missions constituting the PM task. The final stage of training brought all the missions together, at the same time. Instruction panels were displayed on the path taken by the avatar to provide the necessary indications as they went along. Oral assistance from the experimenter was sometimes necessary to provide clarification on these instructions. During the first sequence, participants learned to use the controllers to move around the city. They could move using a touchpad on the right joystick. The avatar, in the virtual city, followed the real rotational movement of the participant’s body. The participant had also a list of places to reach that he could display in front of him by raising the left joystick to eye level. The list was attached to a virtual watch, indicating the time in the environment in minutes and seconds. On the list, there were different places present in the city in a specific order. The goal was to reach these different places by walking, using the traffic signs to navigate, and to validate each mission on the list (Fig. 3). The second training sequence consisted in getting close enough to the place in question (less than 2 m in the VR), waiting for a “beep” that indicated that the item had been checked off the list. On hearing this, a “√” sign appeared (to the left of the element), indicating that the step had been correctly validated. Once validated, participants had to go to the next place using the next signs. The different places were to be followed in the order presented, creating a similar path for all participants through the city. On their way, as a third training sequence, we explained to them that they would have actions to perform in different places, which could come, in particular, from SMS. Then, during the fourth sequence, we added an excerpt from a podcast and questions concerning the podcast; this specific step simulated the ongoing task in the PM assessment. Finally, the fifth and last training sequence consisted in performing all the previous sequences at the same time.

#### PM task

The PM task consisted of a twenty-minute navigation in the virtual city (duration = 20.06 ± 2.33 min). Participants were immersed in the virtual environment and had to carry out the 3 missions. One of them was the PM task. The objective of the navigation task (on-going task N°1) was to complete 7 activities that were part of an original vacation scenario.

The scenario was presented as follows: “*You are leaving on a trip tomorrow and not everything is yet finalized for your stay. You must go to different places in the city, following the imposed itinerary, in order to finalize your preparations. In turn, you will have to go to a clothing store to buy a swimsuit, to the supermarket to take a passport photo, to the city hall to get your passport, to the travel agency to book a night in a hotel, to the pharmacy to buy mosquito repellent and to the bank to buy insurance*”.

The different locations had to be reached in the order presented, creating a similar path for all participants through the city. While completing this set of objectives, participants were asked to listen to a podcast (on-going task N°2). The podcast was an interview of a young boy who went on a road trip with his parents in a motorhome for 6 months. As the participants went along, questions relating to the content of the podcast appeared on the screen in front of the virtual screen and participants had to answer them aloud. The objective was to check that they were focused on listening to the podcast during their navigation. Twenty open questions relative to the podcast content were asked over the entire duration of the navigation (i.e., “*What did Clement prefer in Italy?*”). One point was awarded for each correct answer. A ratio was then obtained by dividing the total number of correct answers by the total number of questions.

The PM task consisted of 24 items to retrieve, 15 encoded before MW induction (see Encoding section), and 9 (3 EBL, 3 EBN, 3TB) which were presented to them during navigation in the virtual city. In the latter case, intentions could come from two sources: from interactions with avatars who walked up to the subjects on their way and who spoke to them, via a written message displayed like a comic bubble; and via the reception of virtual SMS messages also displayed in front of them. A PM item was composed of a prospective temporal cue (the reference time is the navigation time, displayed on the virtual watch) or a prospective environmental cue (places/monuments present in the environment) and an action to be performed associated to this cue. The list of all PM cues is presented in Supplementary Material (Table 1).

#### Navigation

Navigation took place 20 min after the encoding phase. Along the path determined by the items on the list they had to reach in the virtual city, participants had therefore to be able to identify the prospective cues (spatial and temporal) and to recall the actions associated with them. For event-based cues, they had to approach to within 2 m of the place indicated on the list when they thought they had spotted it in the environment. Then, they had to pull the trigger behind their right controller, causing a sign to appear that read “What can I do for you?” Only then could they recall the action out loud and then walk away. For time-based cues, they could simply state the action, aloud, at the appropriate time. In this case, the watch indicated the time and participants therefore had to remember to look at it to check what time it was. The time was not always visually accessible, participants had to raise their left wrist, where the virtual watch was located, to eye level. For each PM item, a standard scoring was used based on our previous VR-PM studies^70,82^: 1 point was assigned if the location or time of action’s retrieval was correct (prospective component) and 1 additional point if the corresponding recalled action was correct (retrospective component). If the two components of the action were correct, the maximum score was 2. Lastly, subjects were submitted to a cued recall of event/time action associations. This was done to control that any possible differences in PM between groups were not due to differences in the PM retrospective component encoding. Of note, all the PM scoring was done by an experimenter blind to the MW manipulation.

### Supplementary assessments

#### Questionnaires

As mood can have an influence on the retrieval of PM intentions, we included the Patient Health Questionnaire (PHQ-4); ^83,84^ as a quick evaluation of depression and anxiety. We also used the MW Questionnaire (MWQ)^85^ a short 5-item questionnaire to evaluate participants’ tendency to experience episodes of mind wandering.

PM abilities strongly depend on the different strategies people use to remember their intentions. In this study, we introduced the short version of the Metacognitive Prospective Memory Inventory (MPMI-s)^80^ that allows for a quick assessment of individual differences in self-reported PM abilities (8 items) as well as in the use of mnemonic strategies (e.g., intention rehearsal, 7 items) and external memory aids (e.g., calendars, 7 items).

Lastly, after VR navigation, participants were assessed on different measures related to the use of VR, such as the Medical Student Stressor Questionnaire (MSSQ)^86^, used to measure susceptibility to motion sickness, the Presence^87^ measuring the degree of presence/experience of the participant in the VR environment, or Cybersickness^88^, assessing whether the VR experience caused the subject to experience cybersickness.

### Procedure

The experiment lasted 93 ± 14 min. Each participant performed the test in the same experimental room to standardize the conditions. They were first asked to read a description of the experiment and to sign a written consent form. Then, the participant completed an anamnesis and continued with 2 questionnaires: MSSQ and PHQ-4 before moving on to 3 cognitive tests: Stroop, Switch and SimAct. Then, the virtual reality training phase was proposed. Once the training was completed and the VR headset had been removed, participants were seated at a desk facing a screen to learn PM items and completed the Metamemory questionnaire. Afterward, participants were confronted with mind wandering induction (n-back task). During this phase, they were left alone in the room to avoid any attentional disturbance on the part of the experimenter who was thus blind to the n-back task. At the end, a second metamemory questionnaire was administered. They were then re-equipped with the helmet and controllers to complete the PM task in VR.

**Figure 4:**
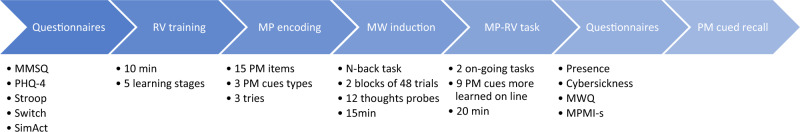
Summary of the experimental protocol.

During the navigation, the experimenter manually rated the accuracy of the PM and on-going task (questions on the podcast content) answers. After a break of a few minutes, giving the subject time to rest from browsing, he was asked to complete the experimentation with a series of questionnaires: post-test metamemory questionnaire, Presence, Cybersickess, MWQ and MPMI -s. Finally, the delayed cued recall was carried out using a dedicated paper form (Fig. 4).

### Data analysis

In order to examine the effects of the experimental condition on the frequency of thoughts, we conducted a mixed ANOVA with Condition (Easy, Hard) as between-subjects factor and the attention Orientation Type (Focus, MW, External) as a within-subjects factor. To investigate the interaction between the experimental condition and the MW orientation content on the frequency of thoughts, we conducted another mixed ANOVA with Condition (Easy, Hard) as between-subjects factor and the MW Content (EPT, EFT, Planning, Imagination and Other) and the MW Source (Spontaneous, Voluntary) as a within-subjects factor. In order to examine the effects of the experimental condition and the type of MP cues on PM performances, we performed two mixed ANOVAs with the Condition (Easy and Hard) as a between-subjects factor and the PM Type (event-based linked—EBL, event-based not linked—EBN, time-based—TB) as a within-subject factor. The first ANOVA was conducted on the cues encoded before the MW task (pre-PM) and the other on those encoded during the VR task (post-PM). Finally, separate multiple linear regressions were performed to predict the scores of each of the three prospective cues, encoded before or after, with the rates of the different components of MW. We reported the effect size for ANOVA with η^2^_p_ (partial eta squared). Post‐hoc analyses were performed using the Bonferroni correction. We used an independent sample (two-tailed) t-test to compare the two groups (Easy and Hard) on demographic, executive, affective and manipulation-check variables. All statistical analyses were conducted with JASP (0.11.1).

## Results

### N-back performances

We reported general lower performances in the Hard group compared to the Easy group. In particular the Hard group was slower to respond to critical t(53) = 3.491, *p* < 0.001 and lures t(57) = 3.669, *p* < 0.001, compared to the Easy group. Moreover, the Hard group had a lower hit rate for critical items t(58) = 3.325, *p* = 0.002. Nevertheless, the Hard and easy group did not differ in either false alarm rate (responding that an item was already presented when it was not) t(58) = 0.126, *p* = 0.9, nor in the discrimination index (A’) t(58) = 1.703, *p* = 0.094. Descriptive statistics are reported in Supplementary Material (Table 3).

We also did not report any significant correlations between the working memory updating scores and MW measured during the n-back task, as well as trait MW (Mind Wandering Questionnaire) neither in the whole sample, nor separately in each subgroup. We reported the detailed results in the Supplementary Material (Table 4).

### Mental states during the retention interval

Second, calculation of the number of MW episodes in relation to the number of thought probes (percentage calculation) showed that, in the EASY group, MW occurred in 32.8% of cases versus 17.8% in the HARD group, t(58) = 3.323, *p* = 0.002. The reliability of our MW task is also substantiated by the observation of a positive relationship between the MW trait, measured by the MWQ, and the MW frequency, measured by the thought probes, r = 0.232, CI [0.076; 0.377], *p* = 0.004. The ANOVA Condition (Easy, Hard) by attention Orientation Type (Focus, MW, External) indicated a significant main effect of the orientation of attention on frequency of thoughts, F(2,116) = 75.717, *p* < 0.001, η^2^_p_ = 0.566. A post‐hoc analysis revealed that the orientation of attention was globally and significantly more Focus (marginal mean = 0.625, 95% CI [0.577; 0.673]) than MW (marginal mean = 0.253, 95% CI [0.204; 0.301]; *p* < 0.001) and External (marginal mean = 0.122, 95% CI [0.074; 0.171] *p* = 0.003). We did not observe a main effect of the Condition Type, F(1,58) = 0.011, *p* = 0.918, η^2^_p_ = 1.847^−4^. Lastly, we revealed an interaction effect between the two factors, F(2,116) = 12.68, *p* < 0.001, η^2^_p_ = 0.179. Post‐hoc analysis showed that participants’ attention was significantly more Focus in the Hard condition (marginal mean = 0.747) than in the Easy one (marginal mean = 0.503, marginal_difference_ = − 0.244, 95% CI [− 0.391; − 0.098], *p* = 0.001) and more MW in the Easy condition (marginal mean = 0.328) than in the Hard one (marginal mean = 0.178, marginal_difference_ = 0.150, 95% CI [0.003; 0.297], *p* = 0.041).

**Figure 5:**
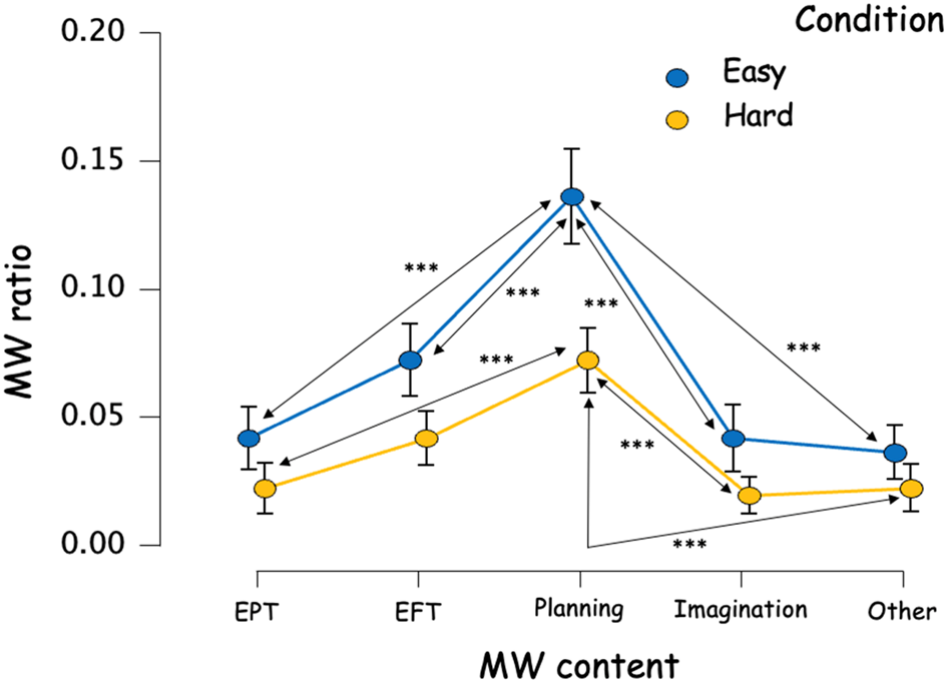
Frequency of different MW contents regarding condition, calculated on all thought probes. ***p < 0.001.

The ANOVA Condition (Easy, Hard) by MW Content (EPT, EFT, Planning, Imagination and Other) on MW frequency showed a significant main effect of the content, F(4,232) = 13.965, *p* < 0.001, η^2^_p_ = 0.194 (Fig. 5). A post‐hoc analysis revealed that the content of the MW episodes was significantly more planning‐oriented (marginal mean = 0.104, 95% CI [0.086; 0.122]) than EFT‐oriented (marginal mean = 0.057, 95% CI [0.039; 0.075]), EPT‐oriented (marginal mean = 0.032, 95% CI [0.014; 0.050]), Imagination‐oriented (marginal mean = 0.031, 95% CI [0.013; 0.049]) and Other-oriented (marginal mean = 0.029, 95% CI [0.0121; 0.047]). The other contents of the MW did not differ from each other. We also found a significant main effect of the Condition Type, F(1,58) = 11.04, *p* < 0.002, η^2^_p_ = 0.160. A post‐hoc analysis revealed that the MW ratio was significantly higher in the Easy group (marginal mean = 0.066, 95% CI [0.053; 0.078]) than in the Hard one (marginal mean = 0.036, 95% CI [0.023; 0.048]). Nevertheless, we did not observe any interaction effect between the MW content and the condition, F(4,232) = 0.892, *p* = 0.469, η^2^_p_ = 0.015 (Fig. 5).

**Figure 6:**
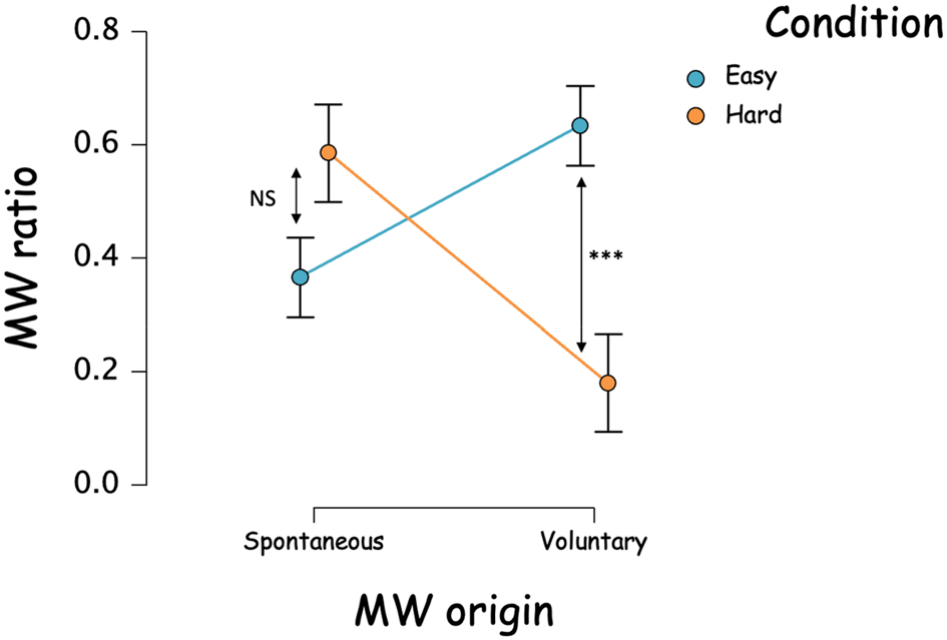
MW source frequency regarding condition, calculated on all thought probes. ***p < 0.001.

The ANOVA Condition by MW Source (Spontaneous, Voluntary) on MW frequency showed no main effect of the MW Source, F(1,58) = 0.761, *p* = 0.387, η^2^_p_ = 0.013. However, we identified an interaction effect between the MW Source and the Condition, F(1,58) = 18.235, *p* < 0.001, η^2^_p_ = 0.239) (Fig. 6). Post hoc analyses indicated that the origin of MW was more voluntary in the Easy group (marginal mean = 0.634) than in the Hard one (marginal mean = 0.181, mean_difference_ = 0.453, 95% CI [0.215; 0.691, *p* < 0.001). Conversely, there was no difference between the two groups regarding spontaneous MW (mean_difference_ = 0.220, 95% CI [-0.458; 0.018], *p* = 0.086).

### Effect of MW induction on VR measurements

We did not observe any effect of the EASY/HARD condition on measures related to VR: feeling of presence (t(58) = − 0.302, *p* = 0.764), cybersickness (t(58) = − 0.526, *p* = 0.601), and time spent in the environment (t(58) = 0.057, *p* = 0.955), thus suggesting no difference on the on-going task N°1. Descriptive statistics are reported in Supplementary Material (Table 5).

### Effect of MW induction on measures of prospective memory

The raw scores of all PM performances are presented in Supplementary Material (Tables 6 and 7).

#### Consistency of prospective memory measure

The two groups did not differ on the on-going task N°2 performances (responses to the questioning about the podcast), with t(58) = 0.634, *p* = 0.528, d = 0.164 (mean_Easy_ = 0.469 ± 0.100, mean_Hard_ = 0.448 ± 0.160). Descriptive statistics are reported in Supplementary Material (Table 5).

#### Effect of condition and cue type on cued recall after the virtual PM task

Regarding PM cues encoded before MW (Pre-PM), the ANOVA Condition by PM Type (EBL, EBN, TB) showed a main effect of the Pre-Cue PM Type, F(2,116) = 34.353, *p* < 0.001, η^2^_p_ = 0.372. Post-hoc comparisons showed that the TB actions were significantly less frequently recalled (marginal mean = 0.840, 95% CI [0.811; 0.869]) than EBN (marginal mean = 0.960, 95% CI [0.931; 0.989], *p* < 0.001) and EBL (marginal mean = 0.994, 95% CI [0.965; 1.024], *p* < 0.001). EBL actions did not differ from EBN (mean_difference_ = 0.034, 95% CI [-0.013; 0.082], *p* = 0.243). We did not observe a main effect of the condition, F(1,58) = 0.524, *p* = 0.472, η^2^_p_ = 0.009, nor an interaction, F(2,116) = 0.622, *p* = 0.539, η^2^_p_ = 0.011.

Regarding PM cues encoded after MW during the virtual task (Post-PM), the ANOVA Condition by PM Type (EBL, EBN, TB) indicated a main effect of the Post-Cue PM Type, F(2,116) = 16.147, *p* < 0.001, η^2^_p_ = 0.218. Post-hoc comparisons showed that the TB actions were significantly less frequently recalled (marginal mean = 0.428, 95% CI [0.363; 0.492]) than EBL (marginal mean = 0.683, 95% CI [0.608; 0.759], *p* < 0.001). EBN actions (marginal mean = 0.533, 95% CI [0.449; 0.618], *p* < 0.001) were significantly less well recovered than EBL (marginal mean = 0.683, 95% CI [0.608; 0.759], *p* = 0.004). TB and EBN actions did not differ (mean_difference_ = 0.106, 95% CI [− 0.004; 0.215], *p* = 0.064). We found a main effect of the Condition, F(1,58) = 4.790, *p* = 0.033, η^2^_p_ = 0.076. Post-hoc comparisons showed that that Easy group had a better retrieval (marginal mean = 0.607, 95% CI [0.531; 0.684]) than the Hard group (marginal mean = 0.489, 95% CI [0.412; 0.566], *p* = 0.033). No interaction was observed between Condition and PM Type on cued recall of Post PM-Cues, F(2,116) = 0.796, *p* = 0.454, η^2^_p_ = 0.014.

#### Effect of condition and cue type on pre PM-cues performances

The ANOVA Condition by PM Type (EBL, EBN, TB) on the total PM scores (expressed by means; maximum score is 2 pts) showed a main effect of the Condition, F(1,58) = 5.996, *p* < 0.017, η^2^_p_ = 0.094. Post-hoc comparisons revealed that Pre-PM performance was significantly better in the Easy condition (marginal mean = 1.294, 95% CI [1.124; 1.464]) than in the Hard condition (marginal mean = 0.999, 95% CI [0.829; 1.170]). We also found a main effect of Pre PM-Cues Type, F(2,116) = 20.528, *p* < 0.001, η^2^_p_ = 0.261. Post-hoc comparisons showed that that the TB actions were significantly less well recovered (marginal mean = 0.920, 95% CI [0.775; 1.065]) than EBN (marginal mean = 1.137, 95% CI [0.992; 1.282], *p* = 0.010) and EBL (marginal mean = 1.383, 95% CI [1.238; 1.528], *p* < 0.001). The difference between the two types of event-based actions was also significant (mean_difference_ = 0.247, 95% CI [0.071; 0.422], *p* = 0.003). However, we did not find a significant interaction between Condition and Pre PM-cues Type, F(2,116) = 0.262, *p* = 0.770, η^2^_p_ = 0.005.

Considering the two components of PM separately (expressed by means; maximum score is 1 pt.), we found for the *retrospective component* a main effect of the Condition, F(1,58) = 4.132, *p* = 0.047, η^2^_p_ = 0.067. Post-hoc comparisons revealed that retro-PM performance was significantly better in the Easy condition (marginal mean = 0.642, 95% CI [0.554; 0.730]) than in the Hard condition (marginal mean = 0.516, 95% CI [0.427; 0.604], *p* = 0.047). We also found a main effect of Pre retro-PM-Cues Type, F(2,116) = 14.096, *p* < 0.001, η^2^_p_ = 0.196. Post-hoc comparisons showed that the TB actions were significantly less well recovered than EBL actions (mean_difference_ = 0.197, 95% CI [0.106; 0.287], *p* < 0.001). EBN actions were also significantly less well recovered than EBL actions (mean_difference_ = 0.117, 95% CI [0.026; 0.207], *p* < 0.007). However, TB and EBN were not different (mean_difference_ = 0.080, 95% CI [-0.014; 0.170], *p* = 0.102). No significant interaction between Condition and Pre retro-PM-cues Type was found, F(2,116) = 0.689, *p* = 0.504, η^2^_p_ = 0.012.

For the *prospective component*, we found a main effect of the Condition, F(1,58) = 6.169, *p* = 0.016, η^2^_p_ = 0.096. Post-hoc comparisons revealed that pro-PM performance was significantly better in the Easy condition (marginal mean = 0.627, 95% CI [0.543; 0.710]) than in the Hard condition (marginal mean = 0.480, 95% CI [0.396; 0.564]). We also found a main effect of Pre pro-PM-cues Type, F(2,116) = 24.709, *p* < 0.001, η^2^_p_ = 0.299. Post-hoc comparisons showed that that the TB actions were significantly less well recovered (marginal mean = 0.420, 95% CI [0.348; 0.492]) than EBN (marginal mean = 0.557, 95% CI [0.484; 0.629], *p* = 0.001) and EBL (marginal mean = 0.683, 95% CI [0.611; 0.756], *p* < 0.001). The difference between the two types of event-based actions was also significant (mean_difference_ = 0.127, 95% CI [0.036; 0.218], *p* = 0.003). However, we did not observe a significant interaction between condition and Pre pro-PM-cues Type, F(2,116) = 0.111, *p* = 0.895, η^2^_p_ = 0.002.

#### Effect of condition and cue type on post PM-cues performances

First, the ANOVA Condition by PM Type (EBL, EBN, TB) on the Total Post PM-Cues performances showed a main effect of the Condition, F(1,58) = 6.184, *p* = 0.016, η^2^_p_ = 0.096. Post-hoc comparisons revealed that Post-PM performance was significantly better in the Easy condition (marginal mean = 1.219, 95% CI [1.086; 1.351]) than in the Hard condition (marginal mean = 0.985, 95% CI [0.852; 1.118]). We also found a main effect of Post PM-Cues Type, F(2,116) = 6.393, *p* = 0.002, η^2^_p_ = 0.099. Post-hoc comparisons showed that the TB actions were significantly less well recovered (marginal mean = 0.956, 95% CI [0.815; 1.097]) than EBL (marginal mean = 1.283, 95% CI [1.142; 1.424], *p* = 0.002), just as EBN were less well retrieved than EBL (marginal_difference_ = 0.217, 95% CI [-0.010; 0.443]) (*p* = 0.044). TB and EBN were not significantly different (*p* = 0.707). Lastly, we did not find a significant interaction between Condition and Post PM cue Type, F(2,116) = 0.220, *p* = 0.803, η^2^_p_ = 0.004.

Considering the two components of PM separately, we found for the *retrospective component* a main effect of the Condition, F(1,58) = 5.343, *p* = 0.024, η^2^_p_ = 0.084. Post-hoc comparisons revealed that Post-PM performance was significantly better in the Easy condition (marginal mean = 0.596, 95% CI [0.524; 0.669]) than in the Hard condition (marginal mean = 0.478, 95% CI [0.405; 0.550]). We also found a main effect of Post PM-Cues Type, F(2,116) = 62.19, *p* = 0.003, η^2^_p_ = 0.097. Post-hoc comparisons showed that the TB actions were significantly less well recovered (marginal mean = 0.506, 95% CI [0.431; 0.580]) than EBL (marginal mean = 0.603, 95% CI [0.559; 0.708], *p* = 0.028), just as EBN were less well retrieved (marginal_difference_ = 0.161, 95% CI [0.044; 0.278]) than EBL (*p* = 0.003). TB and EBN were not significantly different (*p* = 1). Lastly, we did not find a significant interaction between Condition and Post PM cue Type, F(2,116) = 0.540, *p* = 0.584, η^2^_p_ = 0.009.

For the *prospective component*, we found a main effect of the Condition, F(1,58) = 5.577, *p* = 0.022, η^2^_p_ = 0.088. Post-hoc comparisons revealed that Post pro-PM performance was significantly better in the Easy condition (marginal mean = 0.622, 95% CI [0.553; 0.691]) than in the Hard condition (marginal mean = 0.507, 95% CI [0.439; 0.576], *p* = 0.022). We also found a main effect of Post pro-PM-Cues Type, F(2,116) = 8.218, *p* < 0.001, η^2^_p_ = 0.124. Post-hoc comparisons showed that that the TB actions were significantly less well recovered (marginal mean = 0.450, 95% CI [0.375; 0.525]) than EBN (marginal mean = 0.594, 95% CI [0.519; 0.670], *p* = 0.016) and EBL (marginal mean = 0.650, 95% CI [0.575; 0.725], *p* < 0.001). The difference between the two types of event-based actions was not significant (mean_difference_ = 0.056, 95% CI [-0.068; 0.179], *p* = 0.833). However, the interaction between Condition and Post pro-PM-cues Type was not significant, F(2,116) = 0.412, *p* = 0.663, η^2^_p_ = 0.007.

#### Predictors of PM correct ratio

We conducted two backward hierarchical regressions on each PM cue encoded before and after the MW task, separately by PM component (retrospective and prospective), with the following predictors: MWQ, Global MW frequency, Ratio of MW of each Content (EPT, EFT, Planning, Imagination, Other) by Source (Spontaneous, Voluntary). Meta Aptitude (MPMI-s), Depression, Anxiety and each measure of Executive Functions were used as covariates in the regression.

For Pre PM-cues, only the performance for global EBL-cues was significantly and positively predicted by both spontaneous past- and EFT-oriented MW episodes. The prospective component of the EBL-cues was only predicted by spontaneous EFT‐oriented MW thoughts, t(59) = 2.026, *p* = 0.047, 95% CI [− 0.043; 0.672], β = 0.330. The retrospective component of the EBL-cues was only predicted by spontaneous past‐oriented MW episodes, t(59) = 2.327, *p* = 0.024, 95% CI [0.038; 0.506], β = 0.282.

For Post PM-cues, only the retrospective component of the EBL-cues was significantly and positively predicted by voluntary EFT-oriented MW episodes, t(59) = 2.252, *p* = 0.028, 95% CI [0.043; 0.742], β = 0.421.

## Discussion

The goal of this study was to explore the causal role of MW on PM. To do this, we compared the effect of two MW inductions (High and Low MW frequency), during the PM retention phase, on PM performances taking into account cue types and both PM components. Our main findings were two-fold. First, a high MW frequency during the PM retention phase, compared to a low one, was linked with a global better retrieval of PM intentions. Secondly, spontaneous past-oriented MW content predicted better retrieval of the retrospective component of Pre-EBL-PM intentions, while spontaneous EFT-oriented MW content predicted better performance of the prospective component of Pre-EBL-PM intentions. Moreover, the retrospective component of the Post-EBL-PM intentions EBL cues were significantly and positively predicted by voluntary EFT-oriented MW episodes. We will discuss the potential twofold functional role of MW, namely, to consolidate an already programmed intention and to anticipate the future, and more precisely PM intentions retrieval context.

Noteworthy, our results highlighted the consistency of the methods used in this study. Firstly, our results on the different aspects of PM showing that time-based PM was less well recovered, compared to event-based PM, especially those semantically linked, are coherent with previous findings^54,78,89–91^. They thus confirm the validity and relevance of our VR PM task. Secondly, the overall rates of off-task thoughts, including external distractions (MW & External, 35.5%), was close to the percentage reported in the literature (30–45%), although in most of the other studies, no distinction was made between internal and external distractions when computing MW frequency^1–3,26^. Thirdly, we confirmed the MW prospective bias whatever the MW induction: individuals spend more time thinking about the near future (planning) rather than the past^7,24,27,38^.

Regarding our main hypothesis, our results concerning the comparison between High versus Low MW induction substantiate that the condition leading to more frequent MW episodes during a PM retention phase allows a global better recall of prospective actions. An alternative interpretation would be that in the Hard condition, the task difficulty produced retroactive interference, leading to diminished PM performances. Although we cannot completely rule out this hypothesis, recent studies have highlighted that post-encoding demanding tasks (2-back and 3-back) similar to our Hard condition do not interfere with memory consolidation when compared to waking rest^38,79^. Moreover, in our study, post navigation cued recall of PM intentions encoded before MW induction did not differ between the two conditions. Interestingly, the PM retro-EBL (retrospective component) was predicted by spontaneous past‐oriented MW. This prediction did not concern the new intentions encoded during the VR PM task after the MW induction. The impact of spontaneous past-oriented MW content experienced during the PM retention phase on its subsequent retrieval can be understood in two ways. On the one hand, in accordance with the “intention superiority effect”^24,27^, we can argue that PM-EBL delayed intentions had a higher baseline level of activation in working memory and were more accessible to consciousness than any other type of information, stored in long-term memory and not accessible to consciousness. Thus, if we stick to the superiority effect of the representations of unfulfilled intentions^24,27,28,39,40,92^, their supposedly greater accessibility could increase the number of spontaneous thoughts of the upcoming task in the retention phase^43,56–58^. Furthermore, the strength of the semantic link existing between a cue and a prospective action to be carried out spontaneously activates the associated pre-existing semantic networks and thereafter engages reflexive-associative processes in prospective memory retrieval^60^. On the other hand, the fact that our result concerned more especially the retrospective part of PM EBL, thus its content, is in keeping with our hypothesis of the positive impact of past-oriented MW on the (re)consolidation of PM intentions. In this line, several studies have already suggested that past-oriented MW content is associated with the (re)consolidation of older memories, by refreshing previous experiences in mental imagery^48–50^. Thus, in our study, the reactivation of intentions throughout a MW episode, during the PM retention phase, could allow their re-encoding, thus strengthening the intention’s representation and leading to a better prospective intention retrieval.

Regarding our mitigated results about prospective bias, although we reported a predominant quantity of planning-oriented thoughts, called in the literature prospective bias, the latter did not predict PM performances, as we also reported in our recent experience-sampling study^26^. Nevertheless, spontaneous thoughts oriented towards the future, considered as thoughts allowing the projection, anticipation, and elaboration of future events, predicted the successful recall of the prospective component of PM intention encoded before MW. This result illustrates the role of spontaneous episodic future thinking during MW in facilitating PM^82,93–97^. This link-up between EFT-MW and PM-EBL concerned the realization of intentions encoded both before and after the MW session, confirming some previous results about the capacity of thinking and imagining the future execution of intentions to facilitate PM^98,99^, and links between EFT and PM capacities^100–103^. Indeed, a clear distinction has been made between imagining future events (episodic future thinking) and remembering to carry out future intentions (PM). The former is the ability to project into the future, to mentally simulate hypothetical situations and thus to pre-experience future events^100,104,105^. This is a complex process that requires the construction of mental scenes and as such is likely to rely on multiple cognitive processes, including executive control^106^, semantic memory^101,107^and self-projection^108^. While particular attention has been paid to the role of episodic memory in EFT, as it relies heavily on the retrieval of episodic details from past memories that provide background information which is then flexibly recombined to construct new future scenarios^105,109,110^, new observations from experimental, neuropsychological, and neuroimaging studies have also highlighted the pivotal role of semantic memory in providing schemas and meanings for constructing a plausible scenario of a personal event in the future^111,112^. EFT is primarily concerned with relevant distant autobiographical events related to desires, possible selves and future self-concepts. In this it differs from PM, which is specific to the practical activities of daily life and is therefore not specifically concerned with anchoring our personal identity and autobiographical memory. However, these two cognitive functions are linked. In a recent study in patients with semantic dementia^101^, it was reported that at an early stage of dementia, PM was partially impaired as a function of the semantic link involved between cues and intentions, while EFT abilities were impaired as a function of temporal distance. Thus, the deficits were only in event-based linked and distant EFT. In contrast, the other forms of PM as well as near EFT were fully preserved and were significantly correlated. Finally, EFT has been associated with the intention formation phase and improvement of prospective memory retrieval^98^. Indeed, while forming a future intention does not require thinking about that future event in a specific way, imagining when, where, and how the intention will be retrieved, and the action performed, has a beneficial facilitative effect on the execution of those intentions^113–115^.

The two MW induction groups also differed in general on PM performances of cues encoded after the MW induction (during the VR navigation). So, there is probably more to it than the effect on the consolidation phase. This could be due to a carry-over effect, inasmuch as those who do more EFT-MW during the retention phase, also do it during navigation, and if one voluntarily does EFT during navigation when one encounters a new cue, it means that one is probably trying to memorize it by imagining the context of execution. Of course, this is speculative, but it could be tested in a study in which we measure MW just after the presentation of a PM intention to be encoded (thus studying the impact of MW on PM encoding). This would be consistent with the effect of *stimulus-dependent thoughts* on encoding in episodic memory^16^. Indeed, if when the MW is totally disconnected from what the participant is doing at the same moment (*task-unrelated thoughts*^13^), the impact of the MW will be analogous to a divided attention situation and will negatively impact the processing of environmental stimuli (encoding and retrieval of PM intentions). However, when the content of the MW is related more or less directly to certain external stimuli (*stimulus-dependent thoughts*)^13^ it may promote their processing, generating spontaneous associations. Thus, in specific cases where the participant experiences stimulus-dependent thoughts, this associative processing will likely allow for a better encoding of them.

For the effect of voluntary EFT-MW on EBL-PM intentions encoded during the VR PM task, it could be argued that deliberate imagination of the virtual immersion and visuospatial context in which Post PM intention completion will later take place may have been easier for the realization of Post PM intention, by enhancing the encoding and retrieval of the new intended action^116^. This possibility is to be taken into account, given that in our study, subjects were trained in a VR training environment (which resembled the VR PM task) prior to encoding Pre-PM cues and MW induction, so they were able to deliberately imagine walking around a VR city, which may also have increased the encoding of Post-PM cues. La Corte et al. (2021) showed a significant correlation between PM and voluntary near EFT (one week) and found that the evocation of near EFT relied on planned personal events. Thus, the authors highlighted the shared mechanism of PM and voluntary EFT situated within a limited temporal window of self-consciousness. Remembering to carry out future actions and pre-experiencing personal events that are supposed to occur should be explicitly linked to our current goals, planned events, and projects in the coming week. Thus, the strategy of simulating future events, which asks participants to project themselves into the future and imagine experiencing the event or performing the action, thereby reinforcing PM, generally concerns projection into the near future^99,117–119^. It would be interesting to further investigate if the voluntary EFT improves PM differently at the initial phase of the formation of the intention, the retention phase or at retrieval^31^.

According to Cole and Kvavilashvili^120^, most spontaneous future thoughts are “pre-made”. In other words, spontaneous future thoughts are considered as a re-iteration of previously constructed future events in memory, i.e. in our case, PM intentions encoded prior to MW episodes, and therefore based on simple, well-understood, memory processes. This would explain why spontaneous future thoughts occur rapidly, are similar to involuntary memories, and nevertheless predominantly concern upcoming tasks and goals^120^. Lastly, these authors raised the possibility that spontaneous future thinking might be the default mode of imagining the future. To go a step further, it would be relevant to test the (re)consolidation hypothesis by investigating in more detail the content of past-oriented MW, disentangling stimulus-dependent thoughts related to the new encoded material (here PM intentions), association with related autobiographical memory or unrelated autobiographical memory. Along the same line, we would gain insight by separating future-oriented into diverse contents of future-thinking related or unrelated to the new PM intentions, since what is currently unclear in MW is whether instances of participants reporting thinking about the past or future deliberately coincide to near or distant temporal projection^112^. We could also investigate the MW content and source at different moments from encoding to retrieval of PM: during encoding, immediately after encoding, or during all the retention phase and finally during the ongoing PM task, and observe the prevalence of each spontaneous and voluntary MW orientation, *near* or *distant* past and *near* or *distant* future, and their respective influence on different types of prospective intentions, those we have already encoded and those to come in a hypothetical but very probable future, a little like daily life in short.

## Strength, limits and conclusion

As we reported in a recent experience-sampling study^26^, assessing PM using a naturalistic approach is essential since PM is a multiple process par excellence expressed in the flow of daily life to allow people’s autonomy. Virtual reality is an excellent compromise between real-world research, which is very ecological but difficult to control, and classic laboratory experiments, which are highly controlled but far removed from real-world situations^77^. The immersive VR paradigm makes it possible to approach PM in the complex problems of real life (continuous flow of multiple activities and multiple intentions to realize). It involves multisensory integration, which allows the emergence of a sense of presence critical for the similarity of virtual and real-life behaviours^121^. Using this highly immersive VR paradigm, we confirmed the presence of a positive impact of MW on PM performance, with a strong prospective bias, without directly predicting PM performances. However, we reported a predicted link between EFT and the execution of prospective intentions, which supports the hypothesis of an MW system strongly committed to the future. As our previous study highlighted, we found that past‐oriented MW, and more precisely spontaneous MW, positively predicted the retrospective component of PM performances. What was only a speculative hypothesis in our previous study is here experimentally reinforced, this time in a controlled environment, by comparing the effect of MW induction after the encoding of prospective intentions to a control condition (with MW removed) on PM performance. The spontaneous double orientation of the MW (past-future) during the retention phase of the prospective intentions could serve both to reinforce the anticipation and the elaboration of the context in which the intentions, encoded beforehand, will be recalled and the consolidation of the content of these intentions (retrospective component). One of the limitations of the present study is that we do not directly asked participants, during the n-back task, if they rehearsed PM intentions. Since, the Easy group did more mind wandering, it is possible that they also had more chance to rehearsed PM intentions. This could explain the advantage of this group, compared to the Hard group, in retrieving PM intention during the virtual navigation. Future studies should investigate this possibility. Moreover, to move a step further in the assessment of MW content, it will be helpful to ask participants directly about the subject of past or future thinking to test whether it concerns the encoded PM cues. Indeed, Cohen et al.^55^ suggested that the higher the cognitive load associated with the PM intention, the more spreading activation that occurs to potentially associated cues in the environment leading to higher rates of PM-related mind wandering. Thus, this idea that mind wandering is affected by cognitive load, but it depends on the nature of the cognitive load manipulation may be an interesting question to explore in a future study. Another limitation is that we relied on a between-subjects design. Even if we succeed in matching the two groups on variables that are known to influence PM, we cannot completely rule out that interindividual differences could in part explain our results. The choice of the between-subjects design was made for practical reason. Indeed, given the complexity of the VR task, it would have been hard to build two comparable parallel forms necessary for a within-subject design. Nevertheless, we acknowledge the necessity for future studies to replicate our findings in a within-subject design. Lastly, considering the VR device, it will be interesting to use a head-mounted display, including eye-tracking, because it will allow the additional assessment of the dynamic nature of monitoring during the PM VR task^51^.

In conclusion, while the temporal orientation of MW has been repeatedly shown to be primarily focused on the future, whether in the context of planning actions to be performed or projecting into hypothetical situations, the latter being critical for the retrieval of PM intentions, MW seems to have a second functional purpose, in addition to planning future actions: it ensures the continuity in time of old memories (in our case, prospective intentions) by making them regularly go through a re-encoding process. MW is therefore intrinsically linked to the functionality of episodic and semantic memory, since it ensures continuity between the past and the future of the individual. What is currently still unclear, however, is whether instances of participants reporting thinking about the future deliberately coincide predominantly with the process of (deliberately) forming intentions to be carried out in the future, and whether reports of spontaneous future thoughts coincide with simply remembering previously formulated intentions of upcoming tasks or a hypothetical but very probable future, a bit like daily life in short. All these questions remain open, offering future studies the opportunity to address the issue of MW functionality in the specific case of PM.

## Supplementary Information

Supplementary Tables.

## Data Availability

The datasets used and analysed during the current study available from the corresponding authors on reasonable request.
