# Supplementary material for: The benefits of mind wandering on a naturalistic prospective memory task
Source: Sci Rep. 2023 Jul 15;13:11432. doi: 10.1038/s41598-023-37996-z (PMC10349849; doi:10.1038/s41598-023-37996-z)
Supplement: Supplementary file 1 — Supplementary Tables. [file 41598_2023_37996_MOESM1_ESM.docx]

**The benefits of mind wandering on a naturalistic prospective memory task**

Girardeau J.C.^1*^, Ledru R. ^1^, Gaston-Bellegarde A. ^1^, Blondé P.^1,2^, Sperduti M.^1†^, Piolino P.^1†*^

**Supplementary Table 1**. Lists of Prospective Memory Cues encoded before and after (in green) mind wandering induction.


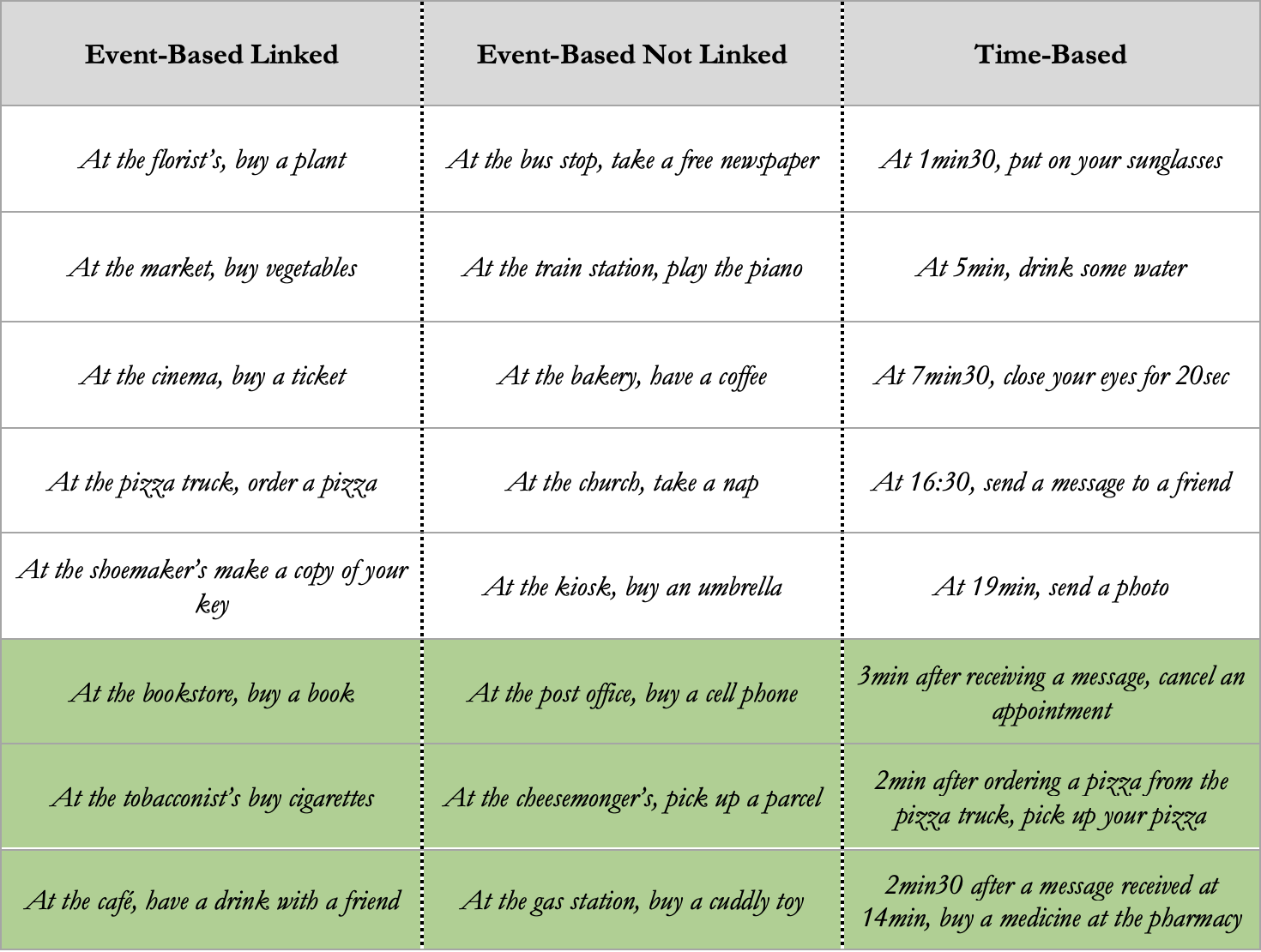


**Supplementary Table 2**. Descriptive statistics of socio-demographic, executive and mood measures.

|  | | **Group** | | **Valid** | | **Mean** | | **Std. Deviation** | |
| --- | --- | --- | --- | --- | --- | --- | --- | --- | --- |
| Age |  | Easy |  | 30 |  | 20.967 |  | 3.469 |  |
|  |  | Hard |  | 30 |  | 22.767 |  | 5.230 |  |
| Education Level |  | Easy |  | 30 |  | 1.133 |  | 1.756 |  |
|  |  | Hard |  | 30 |  | 1.233 |  | 1.832 |  |
| Anxiety (PHQ-4) |  | Easy |  | 30 |  | 5.631 |  | 1.905 |  |
|  |  | Hard |  | 30 |  | 4.613 |  | 2.409 |  |
| Depression (PHQ-4) |  | Easy |  | 30 |  | 3.492 |  | 1.771 |  |
|  |  | Hard |  | 30 |  | 3.319 |  | 2.427 |  |
| Inhibition (Stroop) |  | Easy |  | 30 |  | 30.812 |  | 16.010 |  |
|  |  | Hard |  | 30 |  | 34.048 |  | 14.437 |  |
| Flexibility (Switch) |  | Easy |  | 30 |  | 0.958 |  | 0.023 |  |
|  |  | Hard |  | 30 |  | 0.949 |  | 0.026 |  |
| Updating WM (SimAct) |  | Easy |  | 30 |  | 2.240 |  | 0.686 |  |
|  |  | Hard |  | 30 |  | 2.262 |  | 0.701 |  |
| Metamemory aptitude (MPMI-s) |  | Easy |  | 30 |  | 2.950 |  | 0.646 |  |
|  |  | Hard |  | 30 |  | 3.021 |  | 0.586 |  |
| MWQ |  | Easy |  | 30 |  | 4.300 |  | 0.864 |  |
|  |  | Hard |  | 30 |  | 3.913 |  | 0.750 |  |
|  | | | | | | | | | |
| MPMI-s = Metacognitive Prospective Memory Inventory, MWQ = Mind Wandering Questionnaire  **Supplementary Table 3**. Descriptive statistics of the n-back performances | | | | | | | | | |

|  | | | | | | | | | | | |
| --- | --- | --- | --- | --- | --- | --- | --- | --- | --- | --- | --- |
|  | | **Group** | | **N** | | **Mean** | | **SD** | | **SE** | |
| RT_hit |  | Easy |  | 29 |  | 5232.147 |  | 684.200 |  | 127.053 |  |
|  |  | Hard |  | 26 |  | 6011.664 |  | 961.859 |  | 188.636 |  |
| RT_cr |  | Easy |  | 29 |  | 5023.801 |  | 416.247 |  | 77.295 |  |
|  |  | Hard |  | 30 |  | 5680.834 |  | 873.054 |  | 159.397 |  |
| hit_ratio |  | Easy |  | 30 |  | 0.750 |  | 0.258 |  | 0.047 |  |
|  |  | Hard |  | 30 |  | 0.517 |  | 0.285 |  | 0.052 |  |
| fa_ratio |  | Easy |  | 30 |  | 0.081 |  | 0.194 |  | 0.035 |  |
|  |  | Hard |  | 30 |  | 0.087 |  | 0.198 |  | 0.036 |  |
| A’ |  | Easy |  | 30 |  | 0.877 |  | 0.222 |  | 0.041 |  |
|  |  | Hard |  | 30 |  | 0.772 |  | 0.254 |  | 0.046 |  |
|  | | | | | | | | | | | |

RT_hit = response time for correctly detected target, RT_cr = response time for correctly rejected lures, hit_ratio = ratio of correctly detected target, fa_ratio = ratio of false alarms for lures

**Supplemantary Table 4**. Correlation between working memory updating and mind wandering.

| **Pearson's Correlations – whole sample** | | | | | | | | | |
| --- | --- | --- | --- | --- | --- | --- | --- | --- | --- |
| **Variable** | |  | | **Freq_MW** | | **Updating WM** | | **MWQ** | |
| 1. Freq_MW |  | Pearson's r |  | — |  |  |  |  |  |
|  |  | p-value |  | — |  |  |  |  |  |
| 2. Updating WM |  | Pearson's r |  | 0.086 |  | — |  |  |  |
|  |  | p-value |  | 0.513 |  | — |  |  |  |
| 3. MWQ |  | Pearson's r |  | 0.056 |  | -0.156 |  | — |  |
|  |  | p-value |  | 0.671 |  | 0.235 |  | — |  |
|  | | | | | | | | | |
|  | | | | | | | | | |

| **Pearson's Correlations – Easy group** | | | | | | | | | |
| --- | --- | --- | --- | --- | --- | --- | --- | --- | --- |
| **Variable** | |  | | **Freq_MW** | | **Updating WM** | | **MWQ** | |
| 1. Freq_MW |  | Pearson's r |  | — |  |  |  |  |  |
|  |  | p-value |  | — |  |  |  |  |  |
| 2. Updating WM |  | Pearson's r |  | 0.207 |  | — |  |  |  |
|  |  | p-value |  | 0.272 |  | — |  |  |  |
| 3. MWQ |  | Pearson's r |  | -0.064 |  | -0.120 |  | — |  |
|  |  | p-value |  | 0.738 |  | 0.528 |  | — |  |
|  | | | | | | | | | |
|  | | | | | | | | | |

| **Pearson's Correlations – Hard group** | | | | | | | | | |
| --- | --- | --- | --- | --- | --- | --- | --- | --- | --- |
| **Variable** | |  | | **Freq_MW** | | **Updating WM** | | **MWQ** | |
| 1. Freq_MW |  | Pearson's r |  | — |  |  |  |  |  |
|  |  | p-value |  | — |  |  |  |  |  |
| 2. Updating WM |  | Pearson's r |  | -0.020 |  | — |  |  |  |
|  |  | p-value |  | 0.918 |  | — |  |  |  |
| 3. MWQ |  | Pearson's r |  | -0.016 |  | -0.198 |  | — |  |
|  |  | p-value |  | 0.935 |  | 0.294 |  | — |  |
|  | | | | | | | | | |
| * p < .05, ** p < .01, *** p < .001 | | | | | | | | | |

Freq_MW = frequency of mind wandering measured with thought probes, MWQ = Mind Wandering Questionnaire

**Supplemantary Table 5**. Descriptive statistics of on-going tasks measures.

**
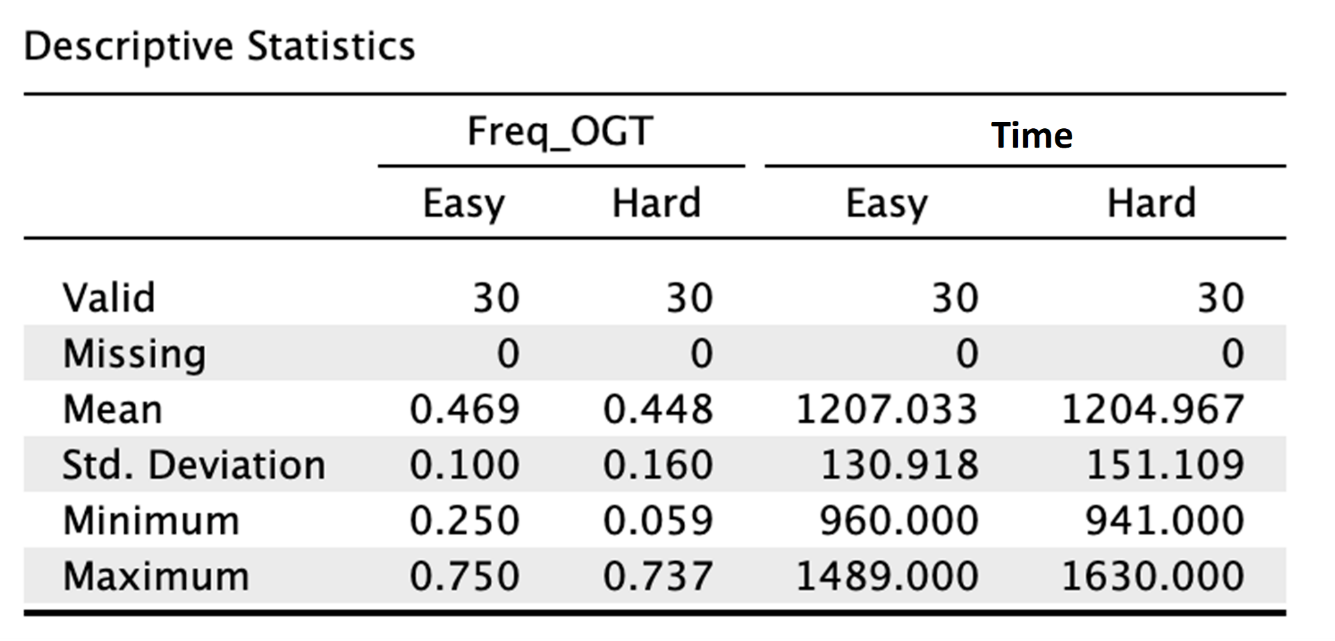
**

**Supplementary Table 6 & 7**. Raw data of all the prospective memory scores encoded before (Table A) or after (Table B) the mind wandering induction.

Table A


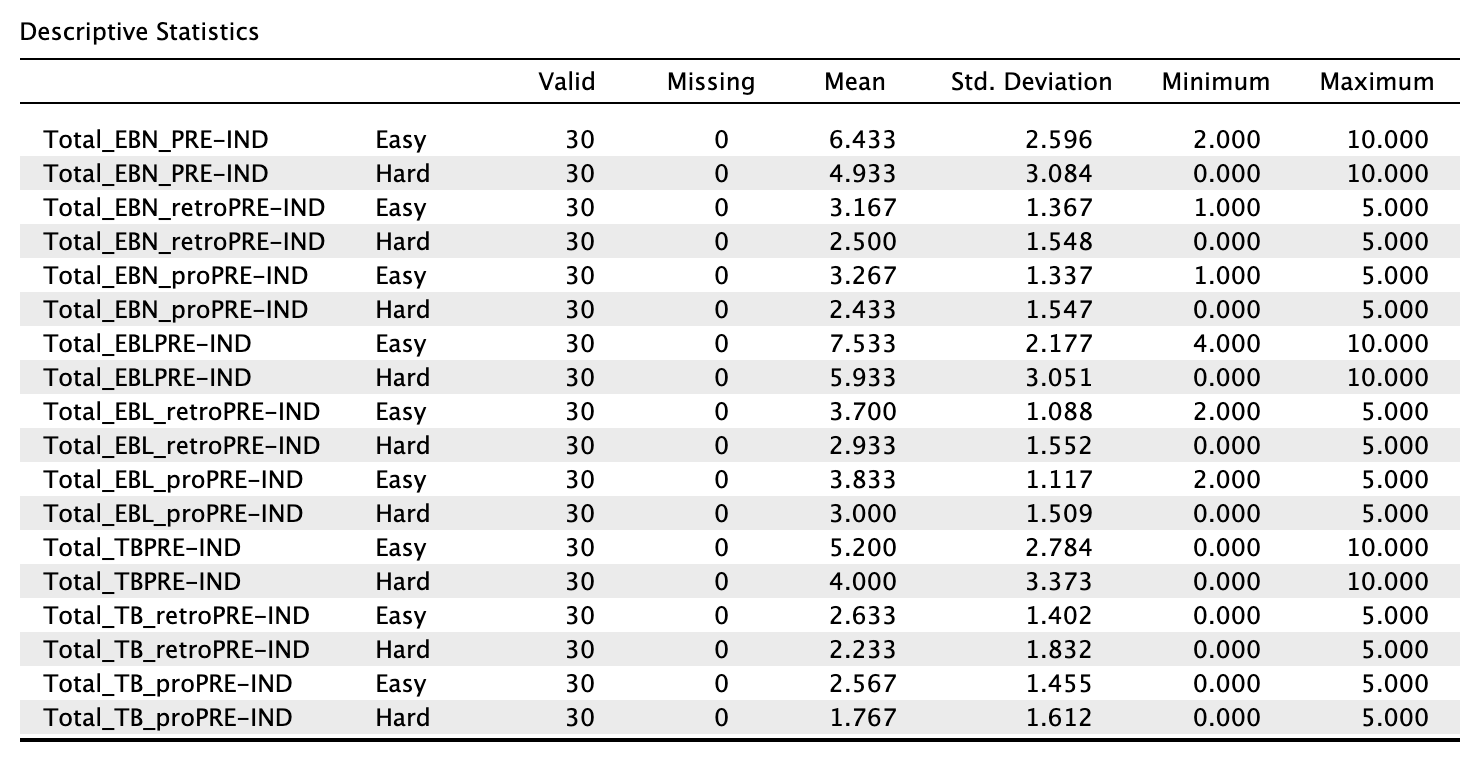


Table B


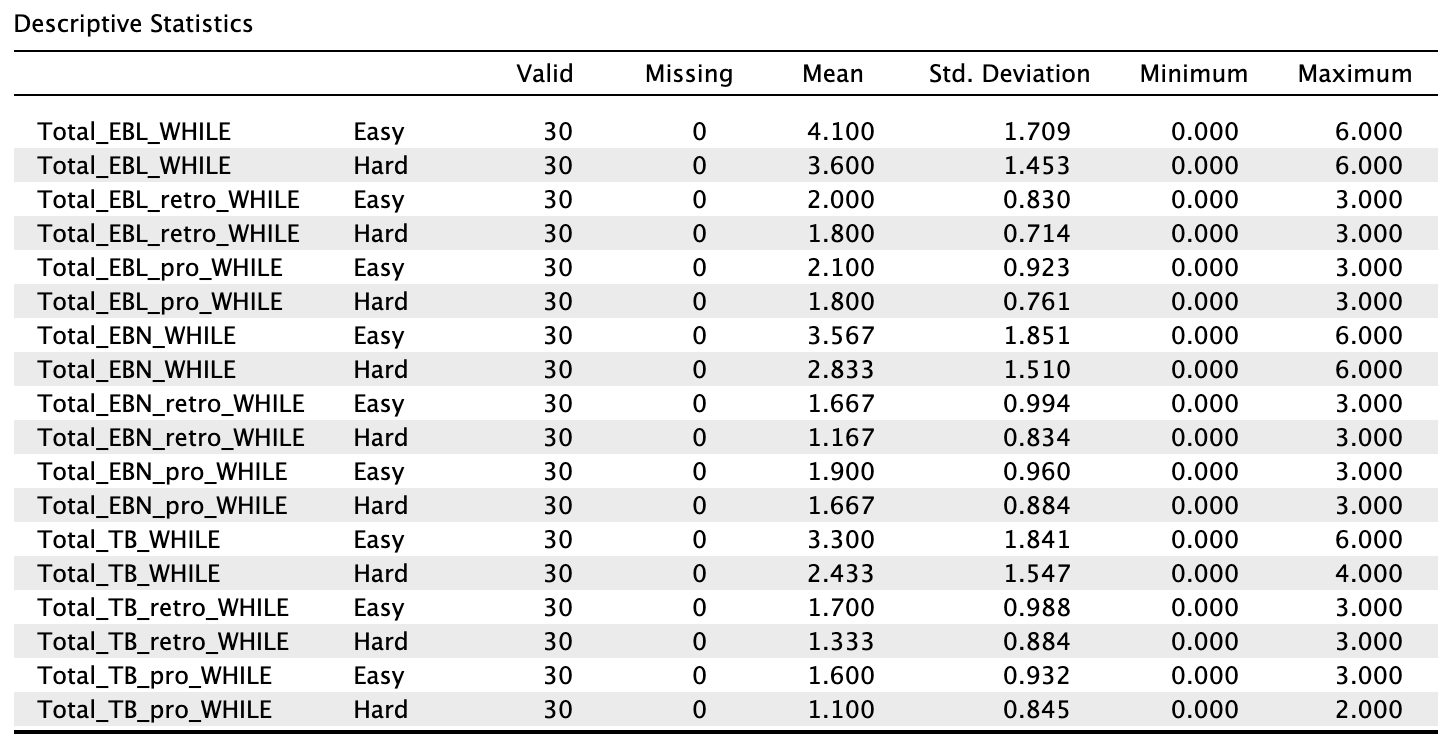


Legend

**EBL_PRE-IND =** *Event-based linked cues encoded before the MW task (pre-induction)*

**EBL_retroPRE-IND =** *Retrospective component*

**EBL_proPRE-IND =** *Prospective component*

**EBLPRE-IND =** *Event-based linked cues encoded before the MW task (pre-induction)*

**EBL_retroPRE-IND =** *Retrospective component*

**EBL_proPRE-IND =** *Prospective component*

**EBN_PRE-IND =** *Event-based not linked cues encoded before the MW task (pre-induction)*

**EBN_retroPRE-IND =** *Retrospective component*

**EBN_proPRE-IND =** *Prospective component*

**TBPRE-IND =** *Time-based linked cues encoded before the MW task (pre-induction)*

**TB_retroPRE-IND =** *Retrospective component*

**TB_proPRE-IND =** *Prospective component*

**EBL_WHILE =** *Event-based linked cues encoded after the MW task (during the PM task)*

**EBL_retro_WHILE =** *Retrospective component*

**EBL_pro_WHILE =** *Prospective component*

**EBN_WHILE =** *Event-based not linked cues encoded after the MW task (during the PM task)*

**EBN_retro_WHILE =** *Retrospective component*

**EBN_pro_WHILE =** *Prospective component*

**TB_WHILE =** *Time-based linked cues encoded after the MW task (during the PM task)*

**TB_retro_WHILE =** *Retrospective component*

**TB_pro_WHILE =** *Prospective component*
